# Institutional Notes and News

**Published:** 1910-09-10

**Authors:** 


### Institutional Notes and News.

**Worthing Hospital.**

The annual general meeting of the Governors of this hospital was held at the Town Hall on Friday, August 26, Colonel Arthur Henty, D.L.J.P., Vice-President, in the chair. The annual report of the Committee of Management, with a statement of accounts, was presented and adopted. This showed a great increase in the number of patients—the in-patients numbering 278, the out-patients 3,195, and 503 minor casualties. It has been found necessary to increase the nursing staff, and the erection of a new out-patient department is also recommended by the committee and approved by the Governors. This would greatly facilitate the treatment of out-patients. The statement of accounts showed a deficit of £276 11s. 7d. on revenue account, and the need of more financial support is strongly emphasised. Although during the past twenty years the work of the hospital has doubled, yet the amount received from annual subscriptions has only increased from £550 to £600. Deep regret was expressed at the loss the hospital has sustained in the death of its President, the late Sir Henry Aubrey Fletcher, who always took so keen an interest in its welfare. The honorary medical staff were thanked for their valuable services, and thanks were also given to the clergy and ministers in Worthing and the surrounding district, to the Salvation Army, and to the various Friendly and Sick Benefit Societies for the valuable financial assistance rendered upon Harvest Festivals, Hospital Sunday, Hospital Saturday, and other occasions. Mr. F. Martin, to the committee's great regret, resigned his position as Secretary and collector at the close of the year, and a resolution was passed appreciating his services, for eight years diligently and efficiently rendered. Captain M. J. Prentice was appointed in his place. A letter was read from His Grace the Duke of Norfolk expressing his willingness to accept the Presidency of the hospital, and in recognition of his services as Secretary, Mr. Frank Martin was elected a member of the committee.

**National Sanatorium for Consumptives, Benenden, Kent.**

Benenden Sanatorium has always been a striking object-lesson in what self-help among workers can do against tubercular disease. The event which will take the Right Hon. Herbert Samuel, his Majesty's Postmaster-General, to Benenden on September 12 is, however, one of the most striking and significant events even in the history of this unique institution. It is, indeed, the climax in the demonstrations afforded by this sanatorium and the movement which it represents. A twenty-bed pavilion has been erected and entirely equipped in every detail by means of subscriptions ranging from the humble penny to sums of 5s. or thereabouts, which have been voluntarily given by Post Office men and women who are members of the Post Office Sanatorium Society. This Society was founded in 1905 and began active work in 1907. Lord Stanley, now Earl Derby, was Postmaster-General when the Society was formed, and helped to forward the movement by every means in his power, and thus worthily continued the traditions of the Derby family in the anti-consumption crusade. When he was succeeded by the Right Hon. Sydney Buxton the latter gentleman was no less sympathetic, and actively supported the Society during the most critical periods of early organisation. The Right Hon. Herbert Samuel, the present occupant of the post, has shown his sympathy by many acts in support of the Society, of which his visit to Benenden will not be the least useful. It is interesting and fitting that the three right hon. gentlemen whose work has so greatly aided the Post Office Sanatorium Society should all be at Benenden when the seal is set on the work of the organisation on September 12.

**What Benenden typifies.**

The entire Benenden scheme is the outstanding feature of the present fight against working-class consumption. The institution as it stands to-day has an accommodation of over 100 beds, and is thus equal in size to the largest sanatoriums in the United Kingdom. It was erected by public subscription, and the members of the working-class societies contributed largely. But the special feature of its finance is the method of maintenance. Every bed is paid for by the workers themselves through the medium of their thrift societies or by means of special societies established for the purpose. The patients are of a class which in ordinary circumstances would resort either to charitable or charity-aided institutions, and thus the sanatorium is relieving in a very practical way the pressure on those institutions. There is, however, no charity taint about the benefits of Benenden Sanatorium; it is self-supporting from funds derived directly from the fellows of the patients and the patients themselves. It is in this feature that the institution is unique, and forms a most valuable object-lesson, not only to the suffering working-classes themselves, but also to the State, which would assist them through the medium of its forthcoming invalidity insurance schemes. The Post Office Sanatorium Society was founded by Mr. C. H. Garland, the present chairman of the sanatorium, and now numbers some 45,000 members, and that large number is daily increasing. Post Office men and women become members of the Society and entitled to all its benefits by consenting to an annual deduction of 2s. from their pay, or less than a halfpenny per week. For this the Society undertakes that when any member is ill with consumption in such a way as to require sanatorium treatment it will find such treatment free of further charge for so long as their medical advisers deem necessary. In addition, and in order to throw the benefits open equally to all parts of the kingdom, the Society pays all travelling expenses incurred by the patients and medical fees for any special treatment incurred during the period when the patient is under its care. The Society maintains permanently thirty-five of the beds in Benenden Sanatorium and keeps other beds occupied in other sanatoriums. It treats about 100 cases a year among its members, and the stay of the patients has in several cases extended to sixty-six weeks, or considerably over a year.

**New Hospital at Rawmarsh.**

The new hospital for infectious diseases at Rosehill, near the Victoria Park, Rawmarsh, has now been opened. The building, which provides for twenty-two beds, has cost £8,000, and, as it will be a rate-supported institution, critics have not been wanting. The County Council at Wakefield, however, has proved firm and energetic, and as Mr. Councillor Bennett remarked at the opening, "if anyone objects to the rates he must not put the cause down to the Urban District Council near at hand, but to the County Council at Wakefield, which was rather more difficult to get at!" The hospital is built on the pavilion system, with the customary lodge and administrative block, and other blocks for scarlet fever, enteric, isolation, discharge, etc. Mr. J. Platts is the architect. Mr. F. Hall, M.P., who lives at Rawmarsh, congratulated the Council on building the hospital, and said that he did not agree that it would prove a white elephant. Miss Oldham, of the Worksop Infectious Hospital, has been appointed matron.

